# An atypical death from Rapunzel syndrome: a case report

**DOI:** 10.1007/s12024-023-00588-4

**Published:** 2023-02-09

**Authors:** Gianluca Nicolò Piras, Luca Tomassini, Edoardo Bottoni, Cira di Gioia, Costantino Ciallella

**Affiliations:** 1 Section of Legal Medicine, Department of Anatomical, Histological, Forensic Medicine and Orthopaedic Sciences, Sapienza University of Rome, Rome, Italy; 2 Department of Radiological, Oncological, and Pathological Sciences, Sapienza University of Rome, Rome, Italy

**Keywords:** Trichobezoars, Rapunzel syndrome, Trichophagia, Forensic pathology, Autopsy

## Abstract

Trichotillomania is a psychiatric disorder characterized by recurring urges to pulling out hairs, eyelashes, or down in other parts of the body. Trichophagia, which is the urge to ingesting the pulled-out hairs, can cause Rapunzel syndrome, an unusual disorder where gastric trichobezoars can be found in the small intestine. Trichobezoars, amorphous masses composed of undigested food formed by hairs, can obstruct the gastrointestinal tract up to simulating symptoms typical of bowel obstruction. Rapunzel syndrome, named after Grimm’s tale, may cause death, especially in the pediatric population, being it seldom over the age of 6; moreover, developing countries and environmental and familiar issues are listed as uncertain risk factors. The present case report deals with the death of a 4-year-old female occurred after lunch and following a series of vomit events; while no traumatic or pathological findings were revealed at the external examination, the autopsy revealed three large trichobezoars localized in the stomach and the small intestine. Despite death was due to gastrointestinal obstruction for multiple trichobezoars and collateral bronchoaspiration of dietary material, histological findings were totally non-specific, meaning that it is sometimes difficult to conclude that death is related to the primary pathological condition.

## Introduction

Trichophagia is described as the urge to repeatedly ingesting hair, generally being one’s own or, anecdotally, other people’s hair, animal fur, hairs found on combs, eyelashes found on tweezers, etc. [1–3].

Hair ingestion, as much as other not edible fibers, can cause complications known as trichobezoars, which are undigested hair masses localized in the gastrointestinal tract, frequently found in children and adolescents [4].

Trichobezoars can obstruct the physiological transit of food bolus, thus causing typical symptoms of bowel obstruction, but they can also result in an atypical gastrointestinal syndrome [5]. This medical condition is referred to as Rapunzel syndrome, an extremely rare disorder due to gastric trichobezoars migration to the small intestine [6, 7].

Diagnosis is frequently difficult to make since the syndrome and its manifestations are sporadic [8]. Rapunzel syndrome, named after Grimm’s tale, can be lethal especially in the pediatric population [9], particularly in those under the age of 6 [4]. The scarce evidence about the disease needs to be attributed to its main occurrence in developing countries and in complicated families [9].

Herein, we present the case of a 4-year-old female, whose death occurred after lunch and following a series of vomit events. The autopsy revealed three large trichobezoars localized in the stomach and in the small intestine; the external examination did not reveal any sign of suspected negligence by the parents.

## Case report

A 4-year-old child was admitted because of vomiting and abdominal pain lasting for 2 days. The mother reported that her daughter had been having issues both in eating and in drinking; the patient appeared dehydrated. According to the mother, it was the first and only episode of these symptoms. The physical examination resulted negative; no pain was reported in abdomen palpation. During the following 5 days, she was clinically diagnosed with metabolic acidosis (i.e*.*, no imaging techniques were implemented) and thus discharged. Blood test showed neutrophil leukocytosis (leukocytes 19.18 × 10^3^/μL, 21.9%; neutrophils 13.29 × 10^3^/μL, 62%; red blood cells 5.74 million/μL). During the short hospitalization, a psychological examination was performed, which resulted in highlighting a symbiotic relationship between the mother and the child, an extremely apprehensive behavior by the mother, who appeared extremely anxious; the neuropsychiatrist noted that when the mother did not intervene in the conversation, the child seemed to be more responsive and less whiny. Further psychological examinations were suggested, in order to prevent any kind of developmental delay, but not performed due to the patient’s scarce compliance. After being discharged, issues in eating and drinking became rarer, though associated with nausea; however, since the symptoms were blurred, patient parents did not seek other medical advice. Two months later, after eating pasta, the child vomited and lost consciousness. Her mother called for emergency services; however, the little girl was already showing clear signs of cyanosis, advanced life support protocols were useless, and she died in a few minutes. A forensic autopsy was performed; the scarce circumstantial evidence was the following: (i) the patient was an only child, and (ii) parents noticed that, sometimes, their daughter ingested her own hair, but, underestimating the behavior, decided for having it cut more frequently.

### External examination

Female dead body, weight 21 kg and length 1.11 m. Oral cavity inspection revealed a non-optimal dental hygiene because of caries and tartar on several teeth; moreover, the left maxillary central incisor (tooth 8 according to the Universal Numbering System) resulted damaged. The girl had fair, 5-cm-long hair, without alopecia areas; eyelashes resulted normally constituted. The body presented some signs of needle injections in the right arm and in the left leg, ECG electrodes, and visible marks of cardiac massage.

Anus appeared patulous; however, when inspecting it together with genitalia no traumatic signs were found, similarly to other body parts.

### Autopsy

The autopsy was performed almost 2 days after the death (46 h); after a 24-h-long period of observation, as required by local law requirements, the body was stored in the morgue for 22 h at an ambient temperature of −10 °C.

The neurocranium, the meninges, and the brain had no macroscopic alterations. The pericardial sac showed hemorrhagic infiltration on its surface due to resuscitation attempts. The heart weight was 85 g and measured 7 × 6 × 4 cm in its diameters, without any sign of malformation or other pathological conditions. The lungs had an increased texture.

**Fig. 1:**
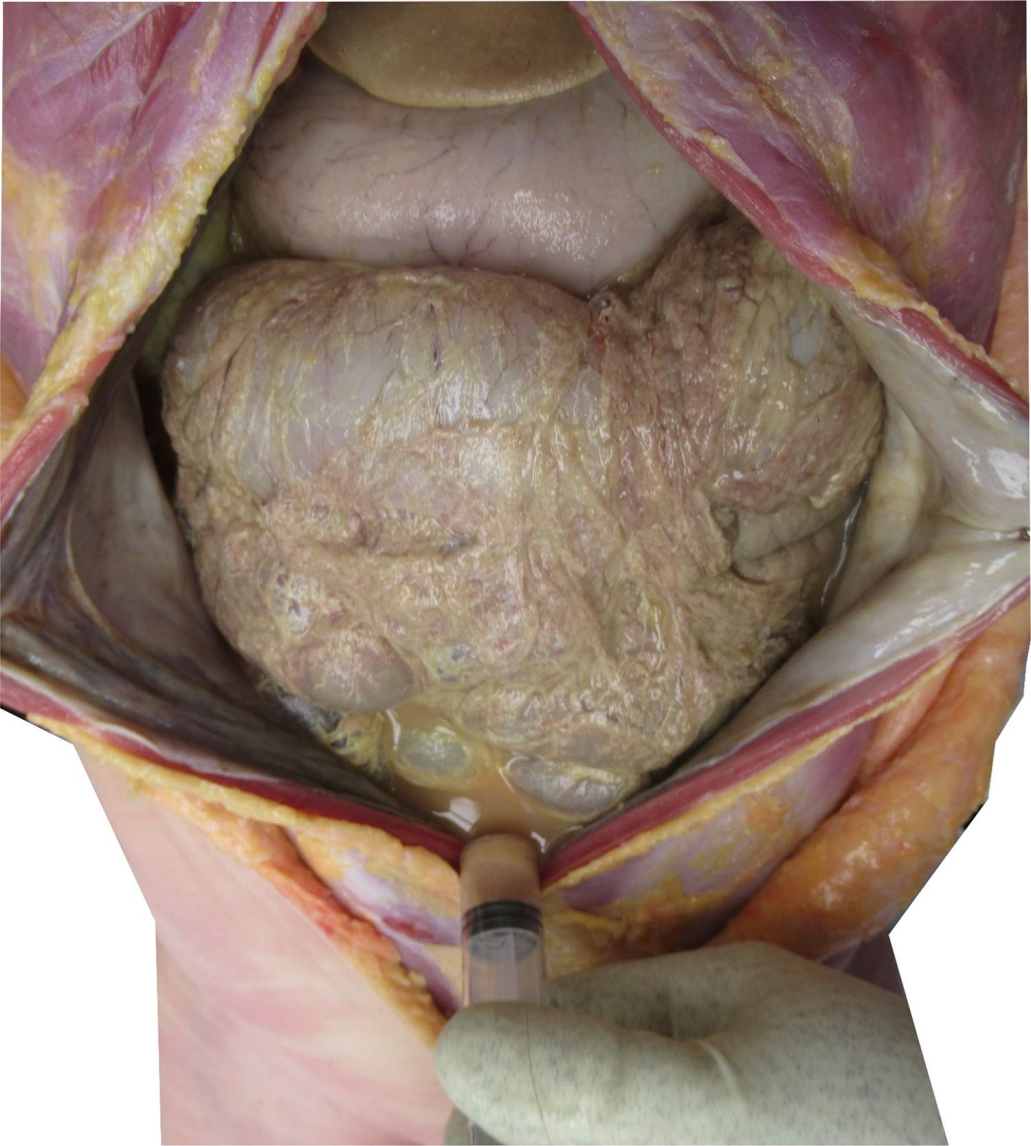
Grey-brownish color of the peritoneum and collecting of the ochre-free liquid found in the peritoneal cavity

The abdominal cavity was filled with about 600 ml of brown free fluid (Fig. 1). Abdominal viscera appeared pale; the parietal peritoneum was opaque and marbled, green-grey in color. No intestinal nor gastric perforation was found.

**Fig. 2:**
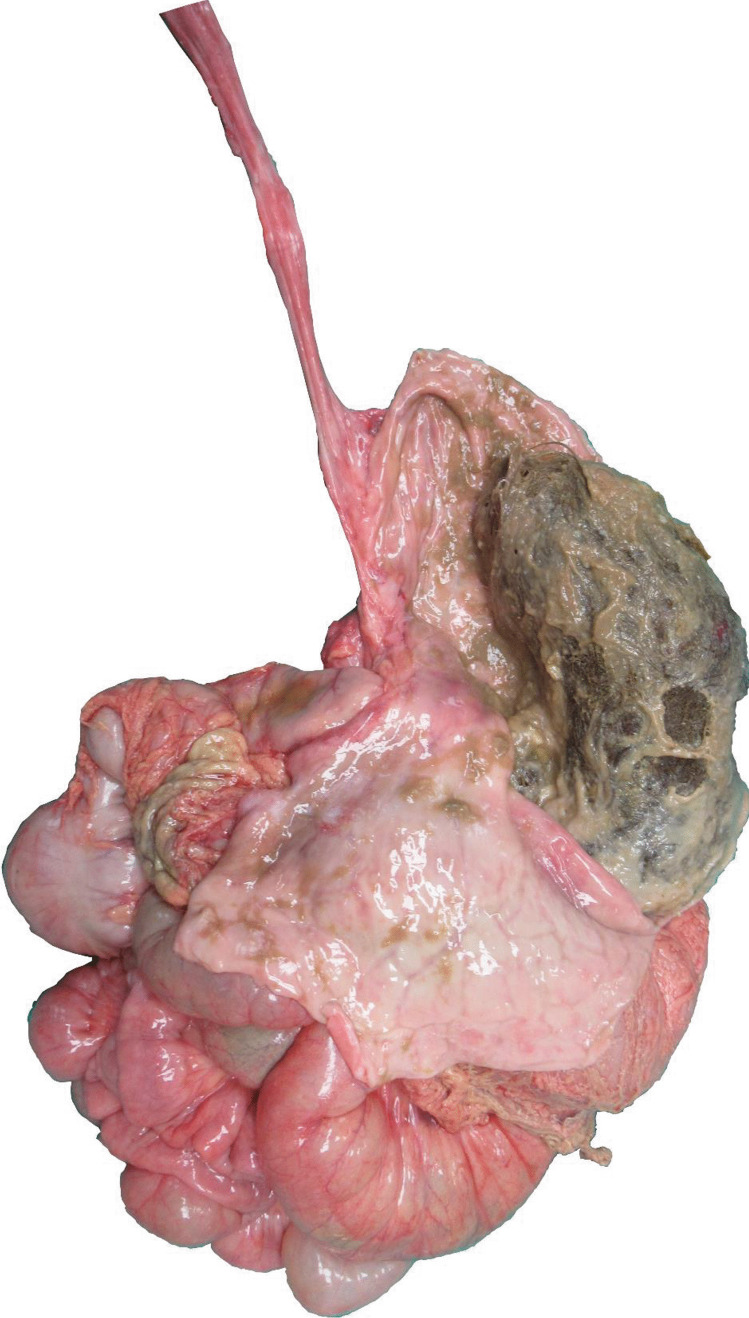
Gastrointestinal tract with the huge trichobezoar found in the stomach

At the inspection and palpation, the gastrointestinal system, the stomach, and the intestine were hard, as in fecal findings. After opening the stomach, a solid, large, brown mass, composed of entangled hairs arose: the trichobezoar was covered by a brown fluid and occupied almost the whole gastric lumen. It weighed 307 g and measured 15 × 11 × 5 cm (Fig. 2).

Other two similar masses were found opening the intestinal loops: the one at the ileo-jejunum section weighed 72 g and measured 9 × 8 × 2 cm, while the one at the ascending and transverse colon weighed 46 g and measured 6 × 4 × 1.5 cm.

**Fig. 3:**
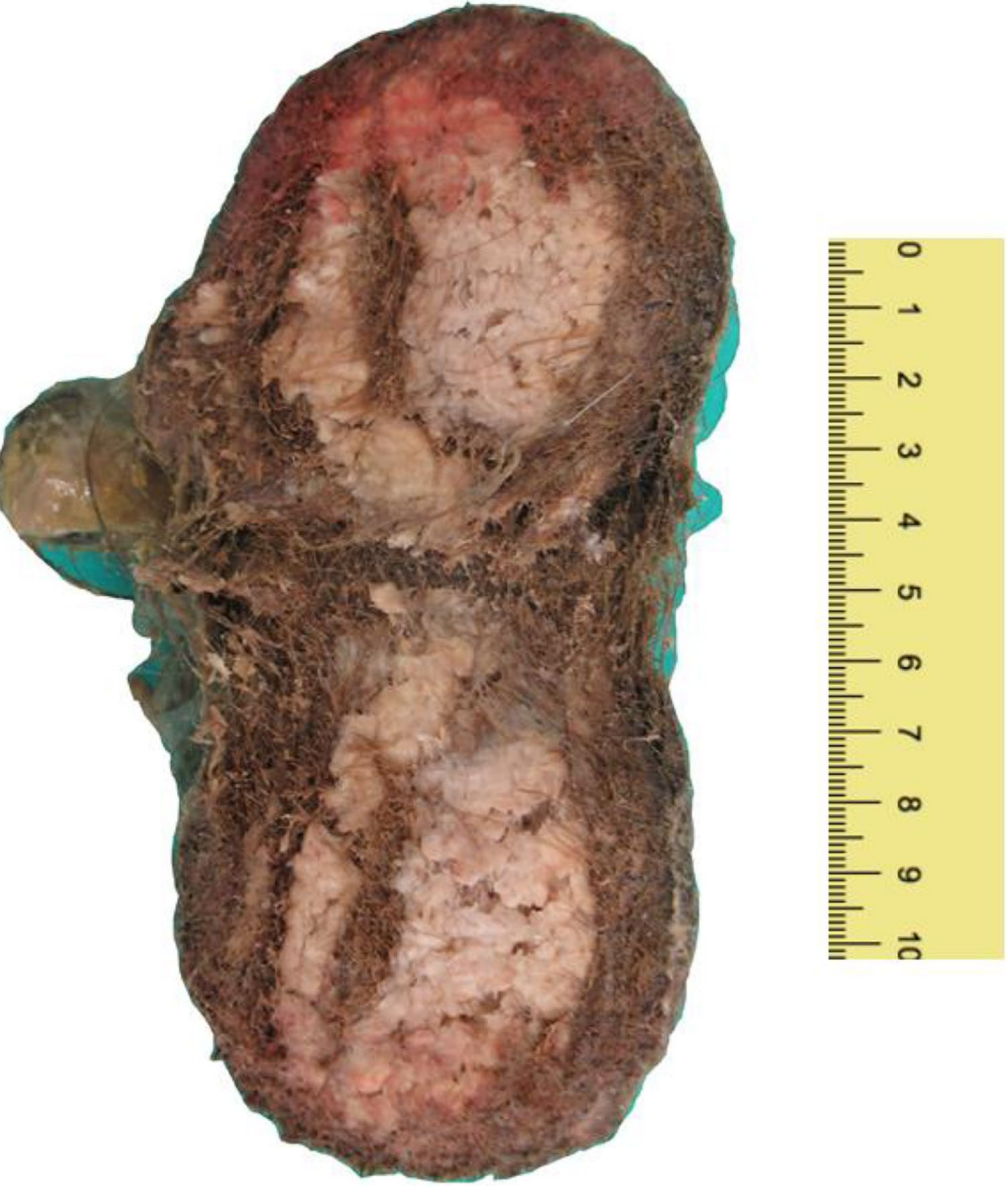
Particular of the section of the gastric trichobezoar. Note the central core, whitish and amorphous, and the wall formed by hairs

After cutting the trichobezoar found in the stomach along its short axis, it showed a sort of internal nucleus composed of amorphous, friable, white material, 7.5 cm in diameter, and an external layer of partially digested and entangled hairs. The other two trichobezoars, once dissected, similarly presented a wall formed by hairs and some focal, whitish spots of amorphous material in the center (Fig. 3).

### Histological examination

**Fig. 4:**
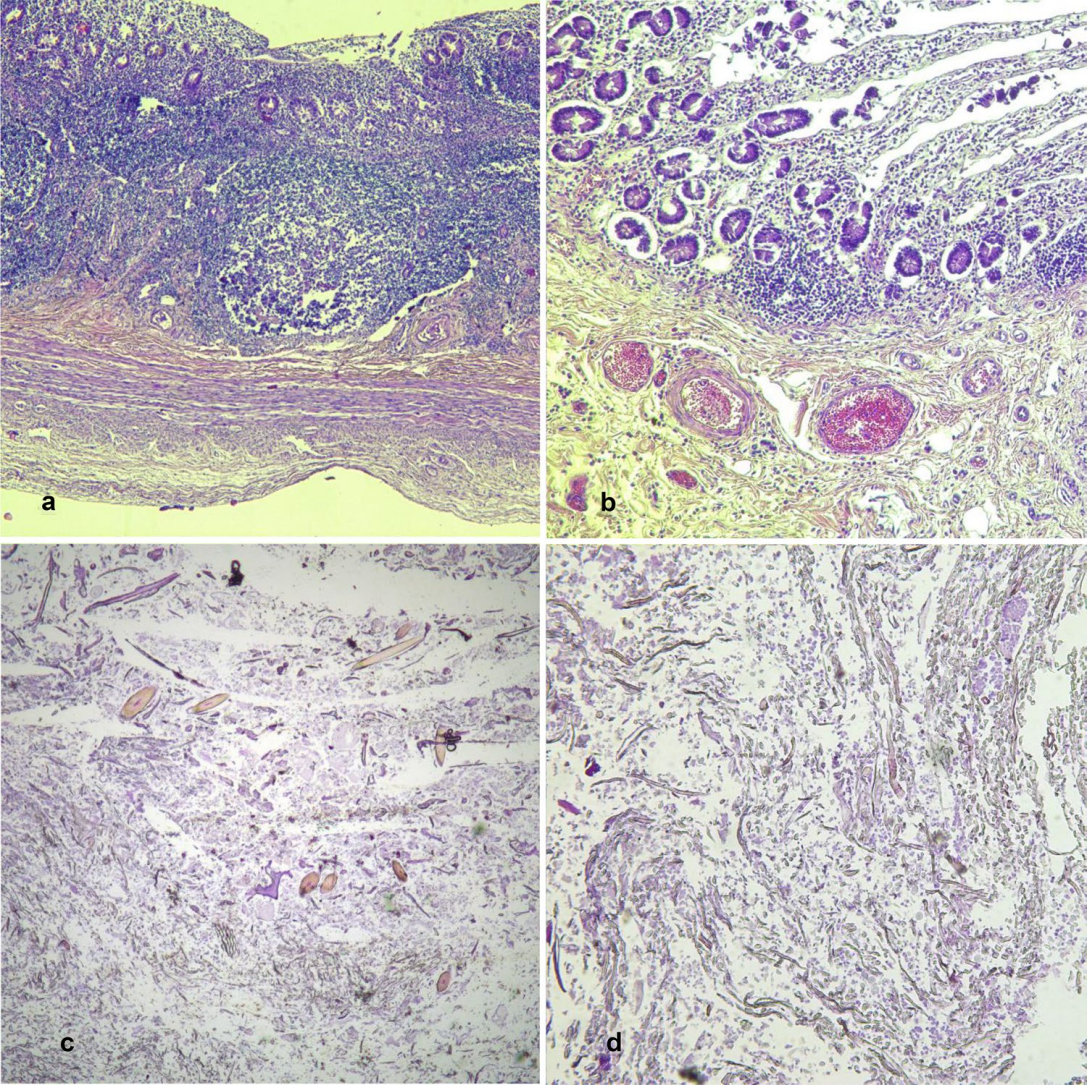
**a** (10 ×) and **b** (5 ×): severe phlogosis of the gastrointestinal wall with inflammation by lympho-monocytes, especially in the tela submucosa and mucosa. **c** 2.5 × : trichobezoar microscopic examination. Section of hairs, dietary fragments (probably vegetables) within amorphous material. **d** 5 × : trichobezoar microscopic examination. Section of hairs

At the microscopic examination, the lungs showed an intense and widespread congestion; dietary material was found in the alveolar spaces and in the bronchial lumen. The pancreas revealed an interstitial inflammatory lymphocytic infiltration consistent with chronic pancreatitis extending to the duodenal wall as chronic duodenitis. The stomach and the small intestine showed chronic lymphocytic inflammation of the mucosa and the submucosa (Fig. 4a, b). Microscopic examination of the heart showed a single hotspot of borderline lymphocytic myocarditis in the interventricular septum. Rectum and vagina had no signs of alteration.

Trichobezoars’ microscopic examination confirmed the dietary nature of the nucleus, specifying it was made of undigested vegetables mixed to hairs (Fig. 4c, d).

**Fig. 5:**
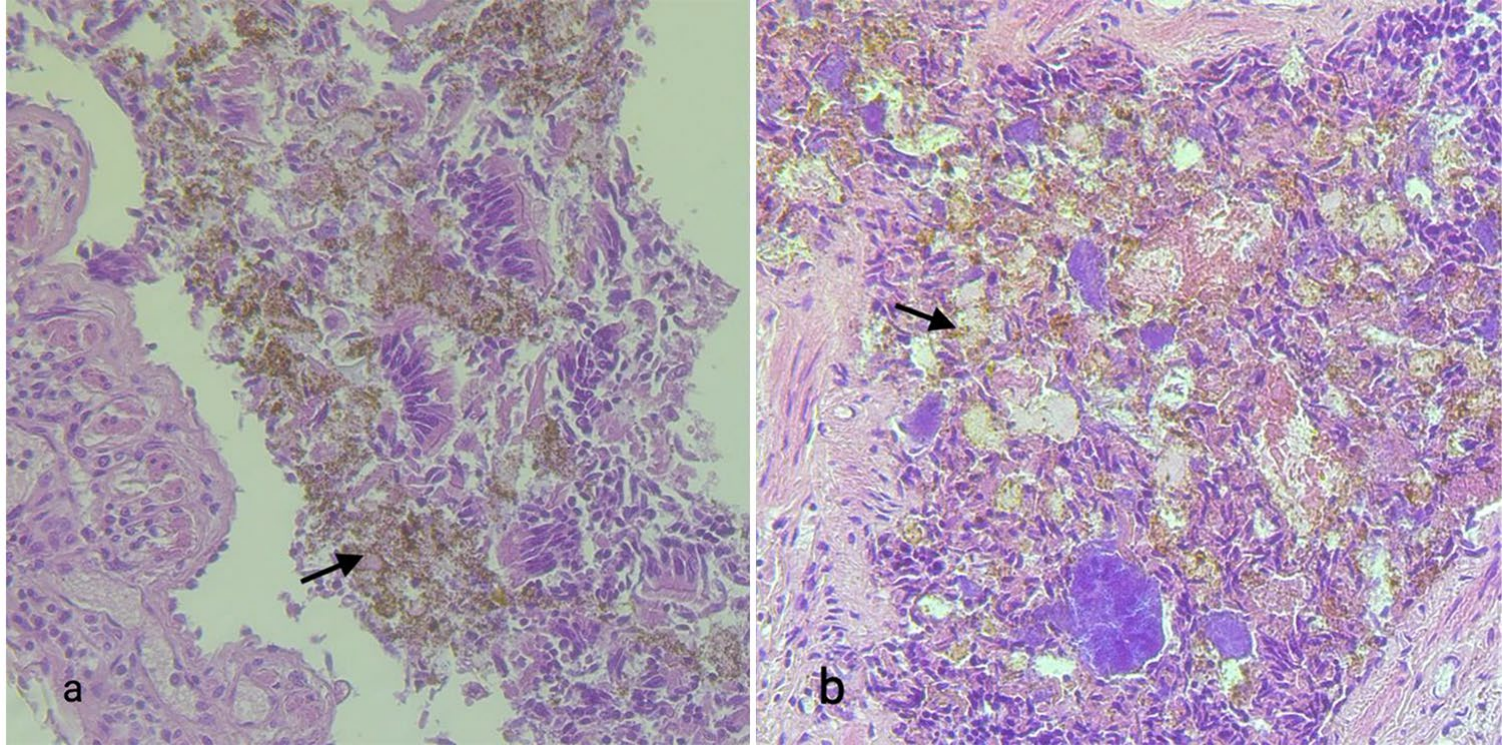
**a** and **b** (20 ×): lung sections showing the presence of bronchoaspiration; arrows point to the amorphous material in the alveoli, where respiratory epithelial cells can be recognized as well

At the microscopic examination, the lungs showed an intense and widespread congestion; dietary material was found in the bronchial lumen (Fig. 5).

## Discussion

As mentioned above, this case is about the death of a 4-year-old girl, whose autopsy found a voluminous trichobezoar in the stomach and two smaller, though still remarkable, ones in the small intestine; leading to the diagnosis of Rapunzel syndrome.

In general, bezoars are masses formed by undigested or partially digested material, which grow in the gastrointestinal tract thus causing obstruction. The incidence rate of gastrointestinal obstruction events due to bezoars ranges from 0.4 to 4% [10]. When bezoars are composed of hairs they are referred to as trichobezoars: this particular kind of mass grows mainly in the stomach, and it occurs in patients with trichophagia, a psychiatric disorder that can affect both children and adults [11].

Women under the age of 20 are the reference population of trichophagia and frequently they have been already diagnosed with other psychiatric disorders, such as anxiety, depression, or ADHD [12]; trichophagia in the pediatric population has been described in low socio-economic classes, in patients with other comorbidities and coming from countries with global development delay [12, 13]. Trichophagia, if chronic and untreated, causes Rapunzel syndrome, which can be considered as the last grade of the disorder, with the bigger trichobezoar which grows in the stomach and then broadens to the small intestine creating its tail(s), particularly in the jejunum and the terminal ileum [14].

Several case reports are no longer available, or they report unverifiable data, whilst it is well documented the case of an almost 4-year-old girl, affected by fetal alcohol syndrome, who died because of bronchopneumonia and malnutrition due to a 210 g trichobezoar in the stomach: social services were alerted for suspicion of neglect [15]. Another case has been reported in Brazil, but data about familiar and clinical status of the child are missing [9].

In our case, the child had been showing gastro-intestinal symptoms for 2 months, in association with nausea and dysphagia/anorexia. Trichophagia was pointed out by the parents, who decided to cut their daughter’s hair more frequently, without seeking any other medical and/or neuropsychiatric evaluation. The family’s socio-economic status was also not known; however, it was said it belongs to the middle class. Since the autopsy was requested by the district attorney, no talking with the parents was allowed, as it would be expected in any other diagnostic process: the scarce information was retrieved from the investigation dossier. The psychiatric evaluation performed during the short hospitalization occurred 2 months prior to the death was deemed inconclusive. Although parental abuse was the main suspicion of the district attorney, the external examination did not reveal any compatible sign; the size of the trichobezoars, though, does imply a chronic hair ingestion which was not confirmed. At the external examination, hair was measured (about 5 cm), which was coherent with the depositions given to the judiciary police.

From a clinical point of view, even if Rapunzel syndrome may cause intestinal perforation and consequently death [16], it entails a wide range of generally non-specific symptoms, such as malabsorption, malnutrition and cachexia, development delay, chronic gastritis, anemia, hypoalbuminemia, intussusception, gastrointestinal, and biliary obstruction [15]. However, it may cause intestinal perforation and consequently death [16]. Moreover, it has been described as causing respiratory distress due to bronchoaspiration, acute peritonitis due to small intestine obstruction, sepsis, multi-organ failure, drug intoxication, and convulsions [15, 16]. In the present case, the only symptoms accused by the child 2 months before the death were abdominal pain and vomiting, which required a brief hospitalization; after being discharged, no symptom was reported, until the cardiorespiratory arrest preceded by after-lunch vomiting. It must be remarked that, during the brief hospitalization, no medical imaging examination was performed, thus trichobezoars, reasonably the cause of the little girl clinical conditions, could not be identified.

As far as the autopsic findings are concerned, neither signs of bowel ischemia nor acute peritonitis were found, given that death occurred almost immediately because of the trichobezoars size; however, the lymphocytic inflammatory infiltration was intense and widespread not only in the gastrointestinal tract but also in the pancreas and in the myocardium. Ultimately, in the absence of other histological findings, the sum of the autopsic findings resulted in declaring the death related to the obstruction due to the three trichobezoars.

The bronchoaspiration identified during the histological examination was related to the vomiting that preceded the death, as testified by the mother. In fact, vomiting is frequently described as a symptom for the presence of trichobezoars [17], although no recurrent vomiting was reported in the present case. Given the considerable amount of the bronchial obstruction caused by the undigested food, even though the superior and medium airways were free, a contributory role in the death was conceded.

The conformation of the trichobezoar found in the stomach was intriguing, given its internal core, amorphous and composed of undigested dietary material, and its cortex formed by hairs. A similar structure was observed in the aforementioned two trichobezoars discovered in the small intestine. It is possible that hairs, once eaten, aggregated around a core of undigested food, such as vegetables which remain longer in the stomach. The other two formations may have grown autonomously in the small intestine or be the detached tails of the gastric trichobezoar, as other Authors suggested [6].

In our case, we hypothesized a combined gastric and duodenal obstruction: since no signs of peritonitis were found, nor intestinal perforation, and given the death speed, our theory was that an endoluminal hypertension occurred first, and an abdominal one later, which compromised the venous and the inferior vena cava return, finally producing a compartmental syndrome. Moreover, in our hypothesis, another mechanism contributed to the physiology of the death, which was a water-electrolyte imbalance; upon this, the episode of vomit right before the death could be another stress factor.

Therefore, our case can be described as an extremely rare death in a young person due to trichobezoars in Rapunzel syndrome. In fact, whereas the autopsic findings are incontrovertible, the intermittent and feeble symptomatology could not clinically prefigure the presence of the undigested hair mixed to food in the gastrointestinal tract. Moreover, the importance of a careful evaluation of non-specific symptoms in the pediatric population is here underlined since no imaging was performed during the brief hospitalization, despite the probable presence of the trichobezoars in the gastrointestinal tract even at that time.

## Conclusion

Given the results of our examination and the literature findings, Rapunzel syndrome is a clinical condition which is hard to diagnose in the pediatric population. At autopsy, findings suggest that large trichobezoars can rapidly cause death without manifesting any typical symptom even for a long time. Histological findings can be non-specific as well—as in this case, there were neither gross signs of acute peritonitis nor intestinal perforation—meaning that death, beyond being quick, is also difficult to relate to the primary disorder.

## Key points

1. Rapunzel syndrome is a complication of trichophagia that can be lethal in children.
2. Symptoms of Rapunzel syndrome can be non-specific and vague, causing death as the first sign.
3. Rapunzel syndrome may occur in unexpected environmental contexts, even in the middle and high classes.
4. Actual cause of death can be unclear even at the histological examination.
